# Technical feasibility and safety of the alternative snare technique using a 0.018-inch guide wire and 5-French catheter for double-J ureteral stent removal

**DOI:** 10.1097/MD.0000000000037525

**Published:** 2024-03-15

**Authors:** Ye Won Lim, Chang Hoon Oh, Hyo Jeong Lee, Soo Buem Cho, Myung Soo Kim

**Affiliations:** a Department of Radiology, Ewha Womans University Mokdong Hospital, College of Medicine, Ewha Womans University, Seoul, Republic of Korea; b Department of Radiology, Ewha Womans University Seoul Hospital, College of Medicine, Ewha Womans University, Seoul, Republic of Korea; c Department of Urology, Ewha Womans University Mokdong Hospital, College of Medicine, Ewha Womans University, Seoul, Republic of Korea.

**Keywords:** percutaneous removal, snare technique, ureteral stent

## Abstract

To evaluate the technical feasibility of the alternative snare technique using a 0.018-inch guide wire and 5-French (Fr) catheter for double-J ureteral stent (DJUS) removal. In this retrospective study, 11 DJUS were removed in 9 consecutive patients between July 2023 and October 2023. We evaluated patient characteristics, DJUS characteristics, and procedure characteristics. Out of 11 cases, 8 (72.7%) were successful in removing the DJUS using the alternative snare technique without major complications. The average time between DJUS insertion and removal was 47.4 ± 50.0 days. The most common DJUS size was an 8-Fr, with proximal tips predominantly in the proximal ureter and renal pelvis. The mean procedure time for successful cases was 15.2 ± 16.8 minutes. Three failed cases, attributed to obstructions like debris, were later successfully addressed using the ALN inferior vena cava filter removal kit, forceps, and modified snare technique. The alternative snare technique using a 0.018-inch guidewire and Fr catheter is safe and effective in cases of DJUS removal.

## 1. Introduction

Cystoscopic guidance with forceps in the retrograde direction is the standard approach to removal or exchange of an indwelling ureteral stent.^[1,2]^ Retrograde retrieval, however, can be difficult or impossible because of proximal migration of the stent, previous surgery on the bladder, the individual anatomic features of the ureter, or enlargement of the prostate.^[3]^ In cases where the retrograde approach is challenging, an antegrade approach can be performed for double-J ureteral stent (DJUS) removal. The overall technical success rate of antegrade DJUS removal is high, ranging from 95% to 100% in previous studies on removal of 26 to 39 ureter stents.^[4,5]^

If the conventional gooseneck snare removal, called simple snare technique fails, using various devices (including forceps, basket, or ALN inferior vena cava [IVC] filter removal kit) can make removal relatively easier.^[4–8]^ Recently, the modified snare technique using a gooseneck snare and a 0.035-inch guidewire has also been widely used.^[9]^ However, the typical snare, that is, the gooseneck snare, has limitations such as a fixed size, high cost, and difficulty in providing torque due to its inability to control direction. Other removal devices, while offering directionality and the ability to grasp the center of the DJUS rather than the tip, can also be expensive and may require a large bore introducer sheath.

In our study, we used a 0.018-inch guidewire and a 5-french (Fr) catheter to create a snare, called alternative snare technique, allowing real-time adjustment of snare size as desired and directionality based on the shape of the catheter tip. We performed an alternative snare technique to remove DJUS. The purpose of this study was to evaluate the technical feasibility and stability of the alternative snare technique.

## 2. Materials and methods

### 2.1. Patient population

This retrospective, single-center study was approved by the Institutional Review Board, which waived the need for obtaining informed consent from the patients (approval no. EUMC 2023-10-013-001). Patients who had undergone antegrade DJUS removal or change at our hospital between July 2023 and October 2023 were enrolled in the study. Patients were 6 men and 5 women with a mean age of 58 years (age range 36–77 years). Nine patients and 11 cases who underwent DJUS were included. Out of the patients, 7 cases underwent radical cystectomy with urinary diversion due to bladder cancer and subsequently had a uretero-ileal anastomosis stricture for which a DJUS was inserted. Two cases, due to cervical cancer, had both a rectovaginal fistula and vesicovaginal fistula, leading to the patients to undergo a total abdominal hysterectomy with bilateral salpingo-oophorectomy, radical cystectomy, and orthotopic neobladder. After surgery, a DJUS was inserted to prevent uretero-ileal anastomosis stricture. Another 2 cases, after undergoing surgery due to cervical cancer, had radiation therapy which resulted in a ureteral stricture. For this result, a DJUS was implemented.

### 2.2. Removal device and technique

For DJUS removal, an antegrade nephrogram was performed to evaluate the hydronephrosis grade, DJUS location, and degree of blood clot retention through previously inserted percutaneous nephrostomy (PCN). Under local anesthesia using lidocaine (Daihan Pharmacy, Seoul, Korea) and fluoroscopic guidance, an 8-Fr introducer sheath (Pinnacle TIF Tip; Terumo, Tokyo, Japan) was inserted over a 0.035-inch hydrophilic stiff guidewire (Radifocus; Terumo) after removing the PCN tube. Neither prophylactic antibiotics nor general anesthesia was given to any of the patients.

**Figure 1.**
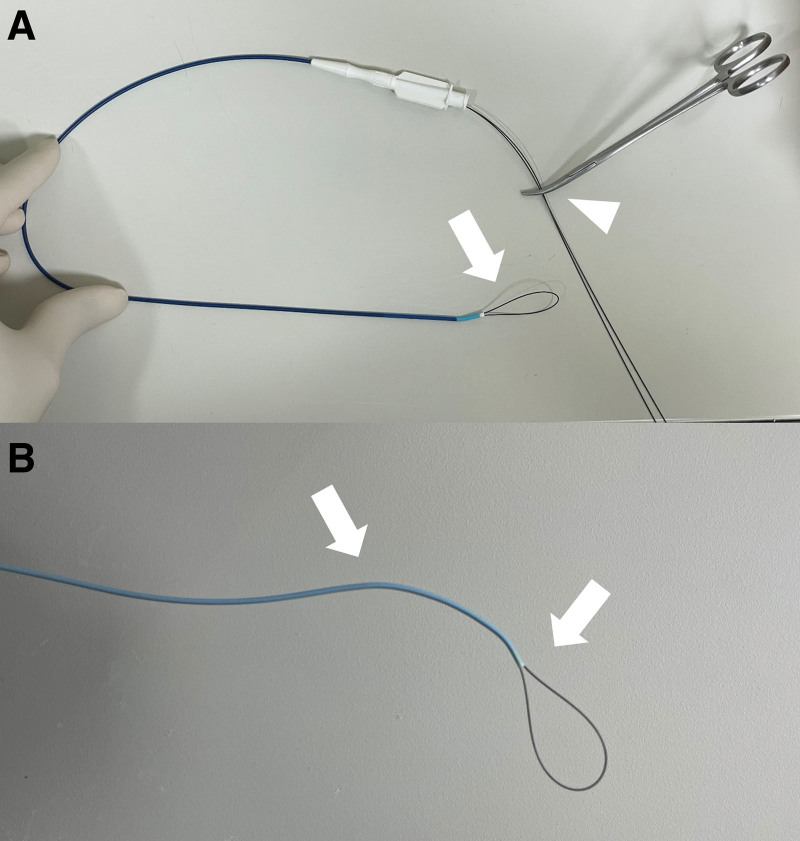
Alternative snare system using 0.018-inch guidewire and 5-french (Fr) catheter. The alternative snare system allows for a loop to be created by inserting a 0.018-inch guidewire into one side of a 5-Fr catheter from the front of the catheter. An alternative snare system was made using a 5-Fr Kumpe catheter (upper), and the back of the catheter was secured with a mosquito (arrowhead) to adjust the size and direction of the loop (arrow). When using a 5-Fr Cobra catheter (bottom) to create the alternative snare system, a larger angle can be given (arrow).

When the alternative snare technique was utilized, following the introduction of the vascular sheath into the renal pelvis via the guidewire, a 0.018-inch guidewire and 5-Fr catheter were inserted through the vascular sheath. On the proximal tip of the 5-Fr catheter, a 0.018-inch guidewire was inserted on each side to form a snare shape. For easier manipulation, the end of the guidewire protruding outside the catheter was secured with a mosquito clamp. Depending on the angle at which the vascular sheath was inserted and the proximal tip position of the DJUS, either a 5-Fr Kumpe or Cobra catheter (Cook Medical, Bloomington, IN) was selectively used. Given the varied lengths of catheters used at our institution, a 150-cm-long 0.018-inch guidewire was used with the 40-cm Kumpe catheter, and a 260-cm guidewire was used with the 75-cm Cobra catheter. In this alternative snare system, if the guidewire was inserted further, the snare-shaped guidewire section in front of the catheter became larger, and if the guidewire was pulled back, the snare-shaped guidewire became smaller. The directionality of the alternative snare system was provided by torqueing either the curved catheter or the mosquito clamp secured at the back (Fig. 1). If the DJUS removal fails with the alternative snare technique, we used a modified snare technique created by combining an additional guidewire, or other devices such as the ANL IVC filter removal kit or forceps.

This alternative snare system, composed of a 0.018-inch guidewire and 5-Fr catheter, was used to grasp the proximal DJUS tip. If the snare-shaped guidewire grasped the proximal tip of the DJUS, the whole assembly, including the DJUS, guidewire, 5-Fr catheter as well as vascular sheath, was removed to pull the DJUS out of the skin. For the patients who required repeated DJUS insertion, a DJUS was inserted through the guidewire in an antegrade direction. If repeated DJUS insertion was not necessary, the vascular sheath was removed or changed with an 8.5-Fr PCN tube.

### 2.3. Study endpoints

In this study, we defined “alternative snare technique failure” as an attempted DJUS removal using the alternative snare technique which was unsuccessful. Technical success was defined as the effective removal of DJUS through alternative snare technique only. Blood clot retention grade was evaluated after the procedure by blood clots in the renal pelvis on a 3-point scale: grade 1, retention of minimal or no blood clots in one or more calyces or the infundibulum alone; grade 2, retention of blood clots in less than half of the renal pelvis; and grade 3, retention of blood clots in most of the renal pelvis and/or ureter. Hydronephrosis grade was divided according to the Onen grade system.^[10]^ Complications were classified as minor or major according to the Society of Interventional Radiology guidelines.^[11]^

### 2.4. Analysis

Analyzed variables and their details were as follows: patient characteristics: age, gender, radical cystectomy with urinary diversion, interval (time to procedure between DJUS insertion and removal); DJUS characteristics: size of DJUS, location of DJUS proximal tip, hydronephrosis grade; and procedure characteristics: procedure time and fluoroscopic dose (mGym^2^), access route (upper, mid, or lower pole of kidney), blood clot retention grade.

## 3. Results

**Figure 2.**
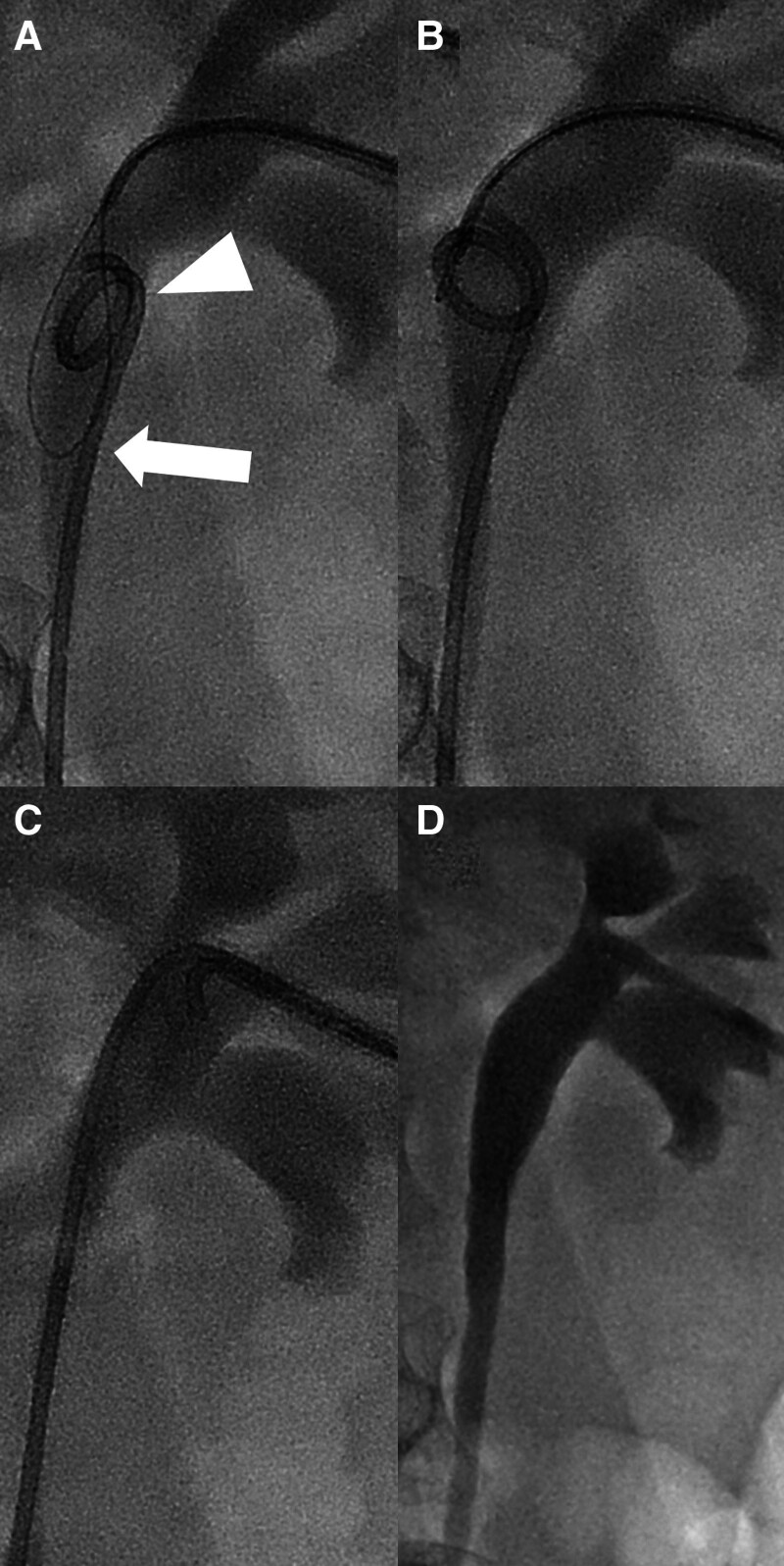
Fifty-eight-year-old man with bladder cancer and undergone radical cystectomy and orthotopic neobladder. (A) After the nephrogram, the alternative snare system was inserted and positioned at the proximal tip of the double-J ureteral stent (DJUS) (arrow). The DJUS proximal tip was located in the proximal ureter (arrowhead). (B) After successful grasp proximal tip of DJUS using proximal loop of alternative snare system, (C) DJUS was removal through 8-french sheath successfully. (D) Final nephrogram showed no definite hematoma within pelvocalyceal system, probable blood clot retention grade 1.

Table 1 summarizes the patients’ clinical characteristics, technical success, fluoroscopic time, hydronephrosis grade before the procedure, blood clot retention grade after the procedure, and follow-up results. Eight of 11 cases (72.7%) were successful in the removal of the DJUS using the alternative snare technique (Fig. 2). Time to procedure between DJUS insertion and removal was 47.4 ± 50.0 days. Eight cases had hydronephrosis grade 1, and 3 cases had grade 2. As for the DJUS size, 6 and 8-Fr stents were the most common (n = 4) respectively, followed by 7-Fr (n = 3). The proximal tip of the DJUS was in the proximal ureter (n = 5), renal pelvis (n = 3), upper or lower calyx (n = 2) and ureteropelvic junction (n = 1). The radiation dose was 378.8 ± 226.8 mGym^2^. The average procedure time for the 8 cases with technical success was 7.0 ± 4.3 minutes.

**Table 1:** Patients’ clinical characteristics, technical success, fluoroscopic time, hydronephrosis grade before the procedure, blood clot retention grade after the procedure, and follow-up results.

Patient no.	Case no.	Age/Sex	Interval (d)	Underlying disease	Hydronephrosis grade	Size of DJUS	Location of proximal tip	Access route	Procedure time (min)	Radiation dose (mGym^2^)	Blood clot retention grade	Technical success	Complication
1	1	M/63	30	Bladder cancer	2	8-Fr	Proximal ureter	Inferior	12	239.8	2	O	X
2	2	M/62	1	Bladder cancer	1	6-Fr	Proximal ureter	Mid	11	298.1	2	O	X
3	3	F/75	4	Bladder cancer	2	8-Fr	Proximal ureter	Inferior	4.5	156.7	1	O	X
4	4	M/71	10	Bladder cancer	2	8-Fr	Proximal ureter	Mid	11	-	2	O	X
5	5	M/77	31	Bladder cancer	1	8-Fr	Renal pelvis	Inferior	1	72.2	1	O	X
6	6	F/36	87	Ureteral stricture	1	6-Fr	Renal pelvis	Mid	8	632.1	1	O	X
	7	F/36	87	Ureteral stricture	1	6-Fr	Ureteropelvic junction	Inferior	42	632.1	3	X	X
7	8	F/44	22	Cervical cancer	1	7-Fr	Renal pelvis	Inferior	8	622.8	2	O	X
	9	F/44	22	Cervical cancer	1	7-Fr	Major calyx of upper pole	Mid	38	622.8	3	X	X
8	10	M/58	167	Bladder cancer	1	6-Fr	Proximal ureter	Mid	1.5	166.3	1	O	X
9	11	M/67	61	Bladder cancer	1	7-Fr	Minor calyx of lower pole	Inferior	15	345.0	2	X	O Pelvicalyceal injury → Improved after PCN placement

DJUS = double-J ureteral stent, Fr = French, PCN = percutaneous nephrostomy.

**Figure 3.**
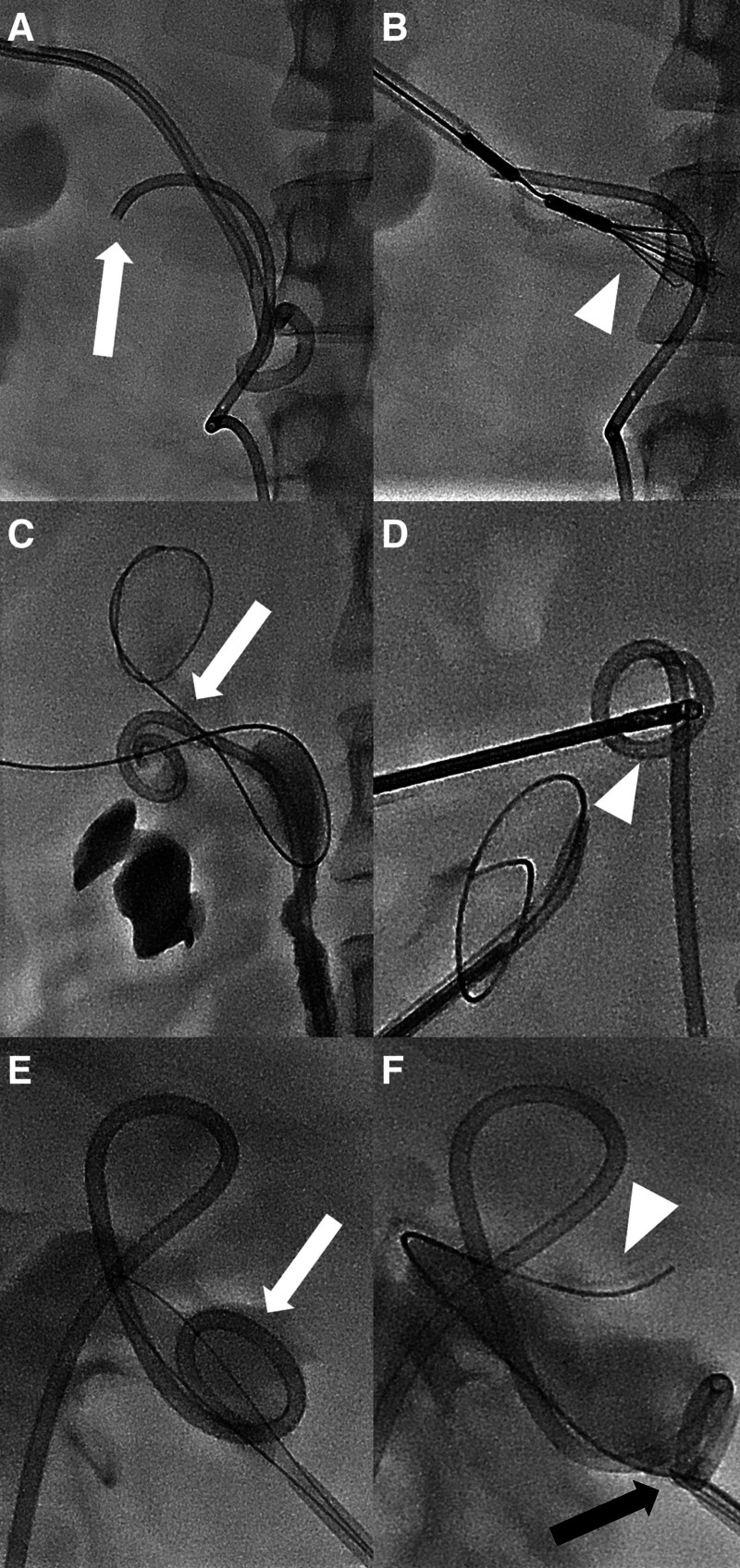
The initial proximal tip of the double-J ureteral stent (DJUS) in the 3 cases that failed and the solution after the alternative snare system failed. (A, B) The DJUS proximal tip was embedded near the UPJ (arrow), so the ALN IVC filter removal kit (arrowhead) was used to capture the shaft of the DJUS and remove it. (C, D) The DJUS proximal tip was located in the upper calyx (arrow), so forceps were used to capture the proximal tip of the DJUS and remove it. (E, F) In a case where the DJUS had proximal migration and the proximal tip (arrow) was stuck in the inferior calyx, an additional 0.035-inch guidewire (arrowhead) was inserted and the modified snare technique was used for removal. During this, instead of the simple snare, the previously used alternative snare system was utilized to capture the 0.035-inch guidewire (black arrow). IVC = inferior vena cava, UPJ = ureteropelvic junction.

The average procedure time for the 3 cases with technical failure was 15, 38, and 42 minutes, respectively. These 3 cases had successful removal of the DJUS through the ALN IVC filter removal kit, forceps, and modified snare technique after alternative snare technique failure. In one of these cases, the patient presented with the urinary system, including the renal pelvis, filled with pus and debris via PCN. The proximal tip of the DJUS was located at the ureteropelvic junction. However, filling defects suspected to be debris were observed around the proximal tip. Due to the pus and debris filling the urinary system, the torque of the alternative snare system was not effective, leading to failure. Ultimately, the removal was achieved using the ALN IVC filter removal kit. In the other cases, the proximal tip of the DJUS was lodged in the upper and lower pole of the kidney, making it impossible to capture with a snare. An attempt was made to snare the tip by puncturing the inferior pole of the kidney and approaching from below upwards, but when it could not be captured, it was successfully removed using forceps and modified snare technique (Fig. 3).

There were no major complications in any of the patients. In patients who successfully underwent DJUS removal using the alternative snare technique and subsequently underwent nephrography, the blood clot retention grade was grade 1 and 2 in 4 cases, respectively. Among the patients who failed, one had a blood retention grade of 2, while the others had grade 3. In one case, a pelvicalyceal injury was observed during the alternative snare technique, so an additional guidewire was inserted, and DJUS removal was performed using a modified snare technique. The patient condition improved after maintaining PCN and he was a few days later.

## 4. Discussion

In this study, for antegrade DJUS removal, we constructed a snare system using only a 0.018-inch guidewire and a 5-Fr catheter, achieving a technical success in 72.7% of cases. Of the 8 patients who underwent successful removal, all presented a blood retention grade of 1 or 2, and there were no major complications in any of the cases. A previous report revealed technical success of 95% to 100% for antegrade DJUS removal.^[4,5]^ However, this finding might have been overestimated, possibly reflecting the use of many removal options, including baskets or forceps. Using only the simple snare technique, success in antegrade DJUS removal was achieved in 69.3% (140/202) of cases.^[9]^ Our results demonstrated comparable outcomes to the simple snare technique. However, further large cohort studies are necessary in the future.

In the study by Mallmann et al, instead of the simple snare technique, they successfully removed intravascular foreign bodies in all 16 cases using a Self-made wire snare.^[12]^ This system was implemented in the same manner as the system used in our study, employing a 0.018-inch and 5-Fr catheter. Although not included in this study cohort, in our institution, there were 3 cases where the through and through wire technique was performed using the alternative snare system when performing percutaneous transluminal angioplasty in arteriovenous fistula patients. In these instances, a retrograde approach was not possible, so an antegrade puncture was performed, advancing the guidewire into the superior vena cava for guidewire capture. All these cases were successfully completed with ease. Compared to the simple snare technique, this method is not size-restricted, and by using an angled 5-Fr catheter, torque can be applied. In situations such as DJUS or foreign body removal, or for rendezvous techniques employing guidewire capture, it appears that the simple snare serves as an adequate alternative. Moreover, it can compensate for the drawbacks of the gooseneck snare, which include high costs and a low rate of usage, making the stock keeping of various snare sizes highly inefficient.^[12]^

In this study, using the alternative snare system for DJUS removal failed in 2 cases where the proximal tip of the DJUS were in the upper and lower calyx. In Kim et al, the location of the proximal tip of the DJUS in the lower calyx (Odds Ratio [95% confidence interval], OR[95%CI]: 18.98 [2.05–175.69], *P* = .01) and in the upper calyx (OR[95%CI]: 33.18 [3.62–303.96], *P* = .002) were independent risk factors in technical failure of the simple snare technique. A proximal tip of DJUS within a confined space, such as the calyces, is unlikely to be captured using the simple snare technique.^[9]^ When we applied the alternative snare technique used in this study, it showed limitations in capturing the proximal tip of the DJUS located within a small confined space such as calyces, like the simple snare (successful rate: 8/11 [72.7%]). Although pushing the guidewire can enlarge the snare shape as desired, it resembles the simple snare technique because enlarging in a limited space is not feasible.

In our other failing case, the removal of the DJUS was unsuccessful due to pus and debris. In this instance, the proximal tip of the DJUS was surrounded by debris, and its tip was embedded, preventing it from being grasped by the alternative snare system. The notion of tip embeddedness as a risk factor for stent removal failure using the simple snare technique is intuitive.^[4]^ According to a prior study, the embeddedness of the DJUS was identified as an independent risk factor (OR[95%CI]: 8.42 [3.98–17.81], *P* < .001).^[9]^ Both the unfavorable position of the DJUS proximal tip and its embeddedness were recognized as risk factors for technical failure for snaring. Recently, there was a case report where a guidewire was inserted into a sheath to create multiple loops for the retrograde approach removal of DJUS.^[13]^ While this method could also be an alternative for the antegrade approach to DJUS removal, it has the disadvantage of not being able to control the torque, and like the simple snare, there are challenges in the small confined space of the pelvocalyceal system, not the urinary bladder. There is also a multi-loop snare called the En-snare, but it may also have limitations in small confined spaces, similar to regular snares. In such cases, DJUS can be easily removed by capturing it not by the tip but by the shaft using forceps, the ALN IVC filter removal kit, or a modified snare technique with an additional guidewire.

In this study, there were cases of pelvocalyceal blood retention or injury among the failed cases. All of these were discharged within a few days without any complications after conservative treatments such as drainage through PCN. According to previous studies, after the simple snare technique or the modified snare technique, there were no major complications, but hematoma, injuries to the pelvis or ureter, and fistulas occurred in 12.4% of cases.^[9]^ In this case, it is believed that due to the relatively long manipulation time in the cases that failed with the alternative snare technique, the grade of the pelvocalyceal hematoma might have increased or injuries might have occurred. However, there were no major complications, and all improved within a few days with treatments like PCN, suggesting that the chance of developing complications is not higher.

There are several limitations to this study. First, it was designed as a retrospective study, which has inherent problems. Second, there was no comparative arm. Third, the number of patients enrolled in this study was small, and additional studies and more cases are needed to further assess the feasibility of this method. Finally, embeddedness of the proximal DJ tip should be evaluated within a cross-sectional image, such as the pelvicalyceal system, which is a 3-dimensional structure. In daily clinical practice, this latter option is not available for most patients and further study might be necessary to determine the accuracy of fluoroscopic evaluation of tip embeddedness.

In conclusion, the alternative snare technique using a 0.018-inch guidewire and a 5-Fr catheter is safe and effective in cases of DJUS removal. However, in cases where there is an unfavorable position of the proximal DJUS tip and tip embeddedness, an alternative removal device or a modified snare technique may be necessary.

## Author contributions

**Conceptualization:** Chang Hoon Oh, Soo Buem Cho.

**Data curation:** Chang Hoon Oh, Myung Soo Kim.

**Investigation:** Chang Hoon Oh.

**Methodology:** Hyo Jeong Lee.

**Supervision:** Chang Hoon Oh.

**Writing – original draft:** Ye Won Lim, Chang Hoon Oh.

**Writing – review & editing:** Chang Hoon Oh.
